# Use of Digital Technology to Fabricate a Metal-Based Maxillofacial Prosthesis for Mandibular Defects

**DOI:** 10.7759/cureus.109157

**Published:** 2026-05-18

**Authors:** Yuan Gao, Mariko Hattori, Yuichi Yamatani, Yuka Sumita, Noriyuki Wakabayashi

**Affiliations:** 1 Comprehensive Treatment Clinic II, Dalian Stomatological Hospital, Dalian, CHN; 2 Advanced Prosthodontics, Institute of Science Tokyo, Tokyo, JPN; 3 Dental Laboratory, Institute of Science Tokyo Hospital, Tokyo, JPN; 4 Partial and Complete Denture, The Nippon Dental University, School of Life Dentistry at Tokyo, Tokyo, JPN

**Keywords:** digital dentistry, intraoral scanner, mandibular defect, mandibulectomy, maxillofacial prosthetics, partial denture, prosthodontics, removable prosthodontics

## Abstract

It is difficult to make conventional impressions for maxillofacial prostheses in patients with complicated anatomical structures. In addition, even after fabrication, multiple adjustments are usually needed due to its complex morphology. This article describes a combined conventional and digital workflow for fabricating maxillofacial prostheses. An intraoral scanner was used to obtain three-dimensional (3D) data on the interim prosthesis and oral cavity of the patient with a mandibular defect. The surface data of the interim prosthesis was 3D printed with acrylic to fabricate an occlusal record base. A metal framework was fabricated using a combination of digital methods for design and wax pattern fabrication and conventional methods for metal casting. An acceptable fit and a satisfactory clinical outcome were demonstrated by the digitally fabricated new prosthesis. Intraoral scanning and 3D printing are useful alternatives to conventional impression and casting techniques for fabricating prostheses for maxillofacial patients.

## Introduction

Mandibular defects can be either congenital or acquired, and the latter may result from surgical treatment for conditions such as orofacial cancer, trauma, and osteonecrosis. These defects can lead to difficulties in mastication and deglutition as well as impaired speech and facial disfigurement [1]. Mandibular resections are categorized broadly as marginal mandibulectomy (partial vertical height resection preserving bony continuity) or segmental mandibulectomy (full-thickness resection disrupting mandibular continuity). The CAT classification system further characterizes segmental defects according to involvement of the condyle (C), mandibular angle (A), and mental tubercle (T), with the AT subtype, affecting the angle and mental tubercle, being commonly observed in mandibular resections [2]. Prosthetic rehabilitation of segmental AT defects is markedly more complex than that following marginal mandibulectomy, primarily due to resultant ipsilateral mandibular deviation, occlusal instability, and associated soft tissue/flap reconstruction deficits [1,3]. 

After mandibulectomy, patients are often fitted with a maxillofacial prosthesis to restore oral functions. The process of fabricating the maxillofacial prosthesis by conventional methods is challenging and requires multiple scheduled visits [1]. Prosthodontists and technicians must also have the skill and experience to address potential challenges such as the risks of aspiration and impaction of the impression material, as well as deformation of the impression caused by conventional fabrication methods [4-12].

Recent studies have shown that it is possible to obtain impressions of both the upper and lower jaws digitally with commercially available devices [4-13]. In addition, computer-aided design and computer-aided manufacturing (CAD-CAM) have been applied to the fabrication of maxillofacial prostheses [4,5,10]. Using digital technology to take impressions and fabricate prostheses is considered safer and more convenient than using conventional methods for these patients [14]. Several studies have reported hybrid workflows incorporating digital technologies for the fabrication of maxillofacial prostheses in patients with maxillectomy [4,5], glossectomy [10], cleft palate [15], or microstomia [16]. However, the workflow for fabricating a prosthesis for patients after mandibulectomy remains unclear.

This report describes the application of a combined conventional and digital workflow to fabricate a maxillofacial prosthesis for a patient with a mandibular defect. The patient was a 68-year-old man with a history of squamous cell carcinoma in the left mandibular molar area that was treated surgically and reconstructed using a graft of scapular osteomyocutaneous flap. The mandibular defect was segmental and classified as AT type according to the CAT classification [2]. After fabricating and adjusting the interim prosthesis, the definitive prosthesis was planned, referring to the structure of the adjusted interim prosthesis. The purpose of this article is to demonstrate the successful use of digital technology to design and manufacture a definitive maxillofacial prosthesis without performing either a preliminary impression or a conventional definitive impression using a custom tray.

## Technical report

**Figure 1:**
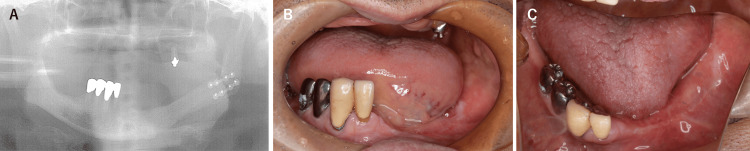
Radiographic and intraoral view of the patient A: panoramic X-ray of the patient's upper and lower jaws; B: intraoral frontal view; C: occlusal view of the mandibular.

**Figure 2:**
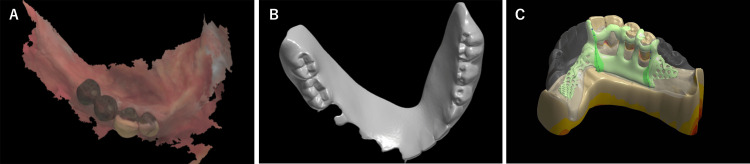
Digital impressions taken using an intraoral scanner and the definitive framework design A: digital impression of the mandibular arch and residual dentition; B: digital impression of the interim prosthesis; C: digital superimposition of teeth and the designed framework.

After a careful examination of the patient’s condition (Figure 1) and appropriate adjustment of the interim prosthesis, an intraoral scanner (Trios 3, 3Shape A/S, Copenhagen, Denmark) was used to capture three-dimensional (3D) data of the mandibular mucosal defect and the residual dentition (Figure 2A). Subsequently, 3D data were also obtained for the interim prosthesis after adjustment (Figure 2B) and the mandibular arch with the interim prosthesis in place. All data were exported as stereolithography (STL) files.

**Figure 3:**
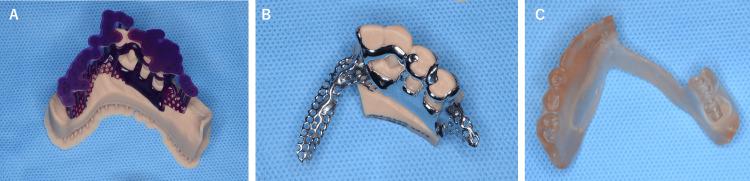
Fabrication of the metal framework and the occlusal record base A: pattern fabricated using a 3D printer; B: framework fitted on a 3D-printed cast; C: occlusal record base fabricated using a 3D printer.

A CAD software (3Shape Dental System, 3Shape, Copenhagen, Denmark) was used to design the removable partial denture (RPD) framework. STL files of the interim prosthesis and intraoral impression were imported to the CAD software. The 3D data of the intraoral impression with and without the prosthesis were placed and superimposed by a best-fit algorithm based on the residual teeth. The data of the interim prosthesis was superimposed by a best-fit algorithm based on the unified data. Then, the necessary components, including the major connector, clasps, rests, proximal plates, and finish line of the metal framework, were designed according to the prosthodontic principle (Figure 2C). A simple circlet clasp was placed on the mandibular right lateral incisor (#42) from the mesial side. Akers clasps were placed on the mandibular right first and second premolars (#44 and #45) from the distal side. A lingual rest was placed on the mandibular right canine (#43). The pattern was printed in wax (Castable Wax, Formlabs, MA, USA) using a 3D printer (Form 3B+, Formlabs, MA, USA) (Figure 3A), and the cobalt-chromium metal framework was cast using the lost-wax technique (Figure 3B).

The occlusal record base was designed based on the 3D data of the interim prosthesis, and it was printed by the 3D printer using UV-curable material (Surgical Guide Resin, Formlabs, MA, USA), which was made of methacrylate monomer, photoinitiator, and urethane dimethacrylate (Figure 3C).

**Figure 4:**
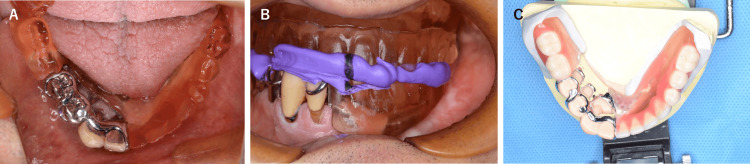
The occlusal record registration and the fabrication of a wax try-in denture A: intraoral view of the metal framework and occlusal record base; B: intraoral view of the occlusal registration; C: wax try-in prosthesis.

The fit of the metal framework was checked, and the framework was bonded to the occlusal record base using auto-polymerization resin (Unifast Pink, GC Corporation, Tokyo, Japan) for occlusal registration (Figure 4A).

After occlusal registration (Figure 4B), the 3D-printed models were mounted on the articulator. The artificial teeth portion of the occlusal record base was sectioned, and the artificial teeth were positioned correctly with paraffin wax (Paraffin Wax, GC Corporation, Tokyo, Japan) according to the arch, using the record base as a reference (Figure 4C). The wax try-in prosthesis was tried intraorally, and the seated impression was taken using a polyvinyl siloxane impression material (Exahiflex Injection, GC Corporation, Tokyo, Japan).

**Figure 5:**
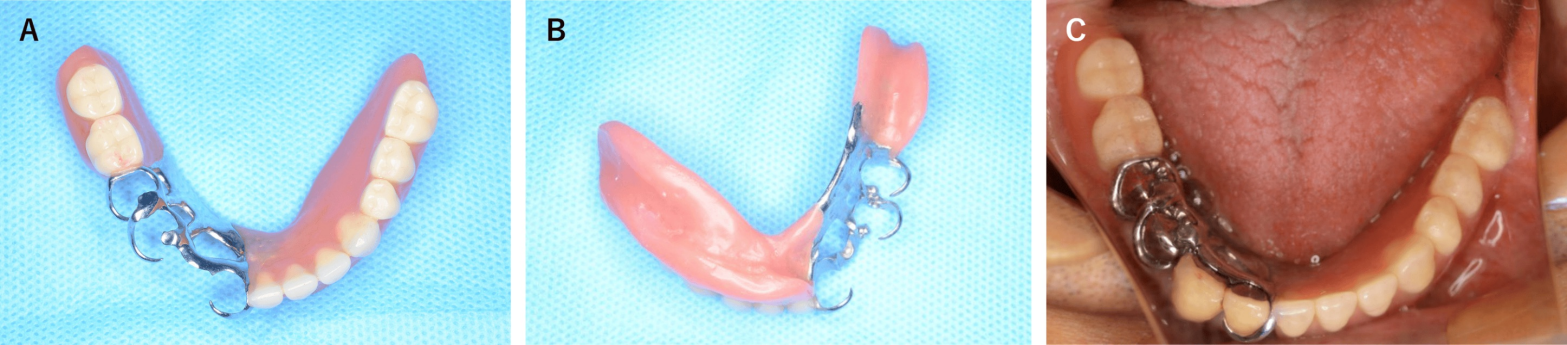
Fabricated prosthesis and the intraoral view wearing it A: occlusal view of the fabricated prosthesis; B: intaglio view of it; C: intraoral view wearing it.

Finally, the definitive prosthesis was processed with autopolymerizing resin (Palapress Vario, Kulzer, Hanau, Germany) in a conventional manner (Figures 5A, 5B). The definitive prosthesis was polished and delivered to the patient (Figure 5C).

**Figure 6:**
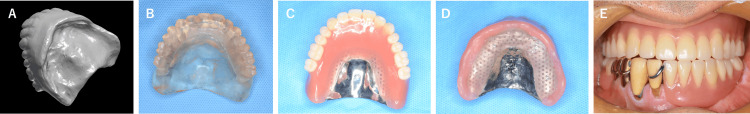
Maxillary prosthesis, which was also fabricated using a digital technique A: digital impression of the well-adjusted maxillary interim prosthesis; B: occlusal record base fabricated using a 3D printer; C: occlusal view of the fabricated prosthesis; D: intaglio view of the prosthesis; E: intraoral view with maxillary and mandibular prostheses in place.

In this case, a definitive prosthesis for the edentulous maxilla was also required; therefore, fabrication of the prosthesis using a digital technique was planned simultaneously. A cobalt-chromium-based complete denture was fabricated in a similar but simpler manner compared with the mandibular prosthesis, as intraoral scanning for designing and fabricating retainers was not required (Figure 6).

## Discussion

The technique described above first uses a digital approach to obtain the impression, fabricate the framework, and design the denture base regions and denture-processing procedures, and then follows conventional protocols for arranging the artificial teeth and fabricating the components at the intermediate stage. The combined use of digital technology with conventional fabrication methods may help to realize more complicated metal framework designs for RPDs for maxillofacial patients with major anatomic structures that have been ablated for tumor removal [4,5,10,13]. Because the interim prosthesis was surveyed digitally, both the preliminary and final impressions were omitted, and the overall process was considered safer and easier to implement than conventional surveying [7,9,12]. In this technique, the digital scan of the well-adjusted interim prosthesis served as a functional impression for distal-extension tooth-tissue-supported areas. A seated impression was then taken to compensate for the surface roughness of the 3D-printed occlusal base and the mucosal surface, resulting in improved adaptation. The occlusal record was transferred to the articulator, and the artificial teeth were manually arranged. Alternatively, the occlusal relationship could be scanned using an intraoral scanner, and the teeth could be arranged digitally in CAD software.

Usually, after making a conventional impression, manual cutting, reduction, stitching, and stone pouring are also required, which can lead to inadequate or excessive trimming and malposition of stitches [10]. There is also the potential for variability in the quality of the dental cast, causing, for example, denture instability, which may occur after the prosthesis is delivered as well [10]. To address this concern, digital registration and stitching were performed by selecting the constant contour features of the residual natural teeth in the scanned model as registration points. In this way, rapid and precise digital construction and more precise revised impressions can be achieved [4,7,14].

The 3Shape Dental System was used to design the RPD framework because of its ability to design complex, well-defined RPD frameworks. The metal framework was designed according to the prosthodontic principle by setting a surveying axis and computing the undercut to determine the optimal path of insertion and removal. Minimal dental preparations were performed according to the design outlined on the digital impression in order to define the outline of the metal bar for the RPD framework underneath the residual teeth for retention and support of the acrylic resin metal bar [5,9,11].

Compared with conventional surveying, digital surveying in the virtual space based on a digital impression can more easily and accurately determine the optimal path of insertion with the desired undercut location [5,7,11]. There are several challenges associated with using digital impressions for removable prostheses, including difficulty scanning certain areas, inaccuracy caused by saliva, and the absence of functional border molding. However, in this technique, these issues are not relevant. This is because digital scanning is performed only for the teeth, while the mucosa and borders are recorded using the well-adjusted existing denture. These problems do not occur in the present method because the soft tissue itself is not scanned; instead, the adjusted surface of the interim prosthesis is used to capture the tissue form.

It could also make it easier to seal unwanted undercuts automatically and select suitable components that can be manufactured based on the digital model [9]. A comparison of the digital framework with the previous design can also be clearly displayed using 3D software. Such a digital workflow has the potential to not only eliminate variability in the quality of the dental cast but also to reduce the time spent in the dental laboratory and clinic. A digitally designed metal framework and denture base can fit the residual teeth and supporting tissue better than a solely conventionally designed framework. The described technique might also help to establish harmonious occlusion and enhance the stability and retention of the prosthesis. As the development of digital technology in the field of stomatology continues to advance, CAD-CAM will be increasingly used as an alternative method for designing and fabricating RPD prostheses in the field of maxillofacial prosthetics.

A limitation of this method is that it requires an interim prosthesis and cannot be applied when fabricating a first prosthesis. In maxillofacial cases, where large or complex defects are present, interim prostheses are commonly used due to the technical challenges of fabricating the definitive prosthesis and the need to accommodate wound healing changes. Surgical stents can also facilitate digital prosthesis fabrication. In contrast, for typical missing-tooth cases without jaw defects, it is common to have a previous prosthesis when the defect is large, as such defects usually develop gradually, starting from one or two missing teeth and progressing to larger areas. Thus, combining conventional and digital approaches allows this method to be applied to a wide range of patients.

## Conclusions

The technique described in this paper provides a combined digital and conventional workflow for fabricating metal-based prostheses for a patient with a jaw defect. By using a well-adjusted interim prosthesis and integrating digital scanning, it simplifies the fabrication process, reduces chair time, and helps coordinate the definitive denture with the previous prosthesis.
